# Estimation of Cell-Type Composition Including T and B Cell Subtypes for Whole Blood Methylation Microarray Data

**DOI:** 10.3389/fgene.2016.00023

**Published:** 2016-02-18

**Authors:** Lindsay L. Waite, Benjamin Weaver, Kenneth Day, Xinrui Li, Kevin Roberts, Andrew W. Gibson, Jeffrey C. Edberg, Robert P. Kimberly, Devin M. Absher, Hemant K. Tiwari

**Affiliations:** ^1^Section on Statistical Genetics, Department of Biostatistics, School of Public Health, University of Alabama at BirminghamBirmingham, AL, USA; ^2^HudsonAlpha Institute for BiotechnologyHuntsville, AL, USA; ^3^Division of Clinical Immunology and Rheumatology, Department of Medicine, School of Medicine, University of Alabama at BirminghamBirmingham, AL, USA

**Keywords:** DNA methylation, whole blood, T cell subtypes, B cell subtypes, epigenetics, deconvolution, cell-type composition

## Abstract

DNA methylation levels vary markedly by cell-type makeup of a sample. Understanding these differences and estimating the cell-type makeup of a sample is an important aspect of studying DNA methylation. DNA from leukocytes in whole blood is simple to obtain and pervasive in research. However, leukocytes contain many distinct cell types and subtypes. We propose a two-stage model that estimates the proportions of six main cell types in whole blood (CD4+ T cells, CD8+ T cells, monocytes, B cells, granulocytes, and natural killer cells) as well as subtypes of T and B cells. Unlike previous methods that only estimate overall proportions of CD4+ T cell, CD8+ T cells, and B cells, our model is able to estimate proportions of naïve, memory, and regulatory CD4+ T cells as well as naïve and memory CD8+ T cells and naïve and memory B cells. Using real and simulated data, we are able to demonstrate that our model is able to reliably estimate proportions of these cell types and subtypes. In studies with DNA methylation data from Illumina's HumanMethylation450k arrays, our estimates will be useful both for testing for associations of cell type and subtype composition with phenotypes of interest as well as for adjustment purposes to prevent confounding in epigenetic association studies. Additionally, our method can be easily adapted for use with whole genome bisulfite sequencing (WGBS) data or any other genome-wide methylation data platform.

## Introduction

DNA methylation is an epigenetic modification that occurs when a methyl group is attached to a cytosine base in the DNA sequence. Methylation typically occurs at sites known as CpGs where a cytosine base is followed by a guanine base. With the introduction of DNA methylation microarrays such as the Illumina HumanMethylation450k (M450), the number of publications on the subject of DNA methylation has increased substantially in recent years. For example, epigenome-wide association studies (EWAS) have been published for traits such as age (Fraga et al., 2005; Rakyan et al., 2010; Horvath et al., 2012; Talens et al., 2012; Day et al., 2013; Hannum et al., 2013), autoimmune disease (Absher et al., 2013; Liu et al., 2013), and lipid measurements (Irvin et al., 2014; Pfeiffer et al., 2015), among others.

In the study of genotyping or DNA sequencing data, the type of cell from which the DNA was obtained is of little consequence. Except for rare somatic mutations, the DNA sequence is the same for all cell types. However, this is not the case for DNA methylation data; DNA methylation varies markedly between cell types (Reinius et al., 2012). Therefore, it is important to account for the cell-type makeup of samples when analyzing DNA methylation data (Adalsteinsson et al., 2012; Jaffe and Irizarry, 2014; Liang and Cookson, 2014). Results of epigenetic association studies (EWAS) can be confounded by cell type if it is not properly accounted for.

Several options are available as ways to adjust for cell-type makeup of samples. If cell-type percentages have been estimated directly (such as in a complete blood count), these measurements can be included in models as covariates. However, these measurements are often unavailable. Principal components often correlate with cell-type makeup (Irvin et al., 2014), so adjusting for a few principal components in a model is often adequate. Other methods that do not directly estimate cell-type percentages are available (Leek and Storey, 2007; Teschendorff et al., 2011; Houseman et al., 2012; Zou et al., 2014). However, it is often useful to obtain estimates of cell-type percentages in order to test for associations with percentages of specific cell types.

Many authors have predicted cell-type percentages using gene expression data (Lu et al., 2003; Wang et al., 2006; Abbas et al., 2009), and some have used similar approaches for DNA methylation data (Houseman et al., 2012; Koestler et al., 2013; Jaffe and Irizarry, 2014). The general method is based on a linear model of the form *B* = *AX*, where *B* represents the gene expression or DNA methylation profile of a mixed sample comprised of several different component types, *A* represents a matrix containing the gene expression or DNA methylation profile of sorted cells of the types making up the sample described in *B*, and *X* is a vector of mixing proportions that describes what proportion of the sample in *B* can be attributed to each of the types in *A*. The expression or methylation profile of the mixed samples in *B* and the purified cell types in *A* are obtained through separate experiments, and a subset of genes or CpGs that are differentially expressed/methylated within different cell types is selected for inclusion into the model in order to estimate the unknown mixing proportions *X*. Lu et al. (2003) used this method to determine the proportion of yeast cells at different phases in the cell cycle based on gene expression microarray data. The authors used optimization by simulated annealing (Kirkpatrick et al., 1983) to mathematically determine the values of mixing proportions that would best satisfy the system of equations without using a linear regression model. Abbas et al. (2009) introduced an error term, creating a linear regression model that could be used to obtain estimates of cell-type mixing proportions through least-squares estimation. The authors restricted the coefficient estimates to be positive so that negative estimates of proportions could not be obtained.

The most commonly used method to predict blood cell-type components using DNA methylation array data is a model proposed by Houseman et al. (2012). This method is based on linking two regression models, one for the purified cell type components and one for the whole-blood samples. Jaffe and Irizarry (2014) published an adaptation of the Houseman method for use on Illumina M450 array data as opposed to the older Illumina HumanMethylation27k array that was used in the original Houseman publication (Houseman et al., 2012). The model is the same, and the method only differs in the set of CpGs that were chosen for inclusion in the model. Although these models provide useful techniques for estimating overall proportions of T and B cells among others, the methods do not provide a way to estimate subtypes of T and B cells. This would be a useful addition to many researches, particularly those studying immune cells and autoimmune diseases.

We propose a two-stage model, based on an extension of the model used by Abbas et al. (2009), for DNA methylation data on whole blood samples. With this model, we are able to estimate the percentage of six different main cell types that make up whole blood. We refine the estimates for similar cell-types, such as CD4+ T cells and CD8+ T cells, using a two-stage model. Most importantly, our model is also able to refine estimates into subtypes of several cells including CD4+ T cells (memory, naïve, and regulatory), CD8+ T cells (memory and naïve), and CD19+ B cells (memory and naïve). The estimation of T and B cell subtypes represents an additional functionality that is not available with currently existing methods.

## Materials and methods

In order to build a model to deconvolute DNA methylation data from whole blood samples to determine the proportions of cell types and subtypes, we used data from Illumina HumanMethylation450k (M450) arrays from individually sorted blood cell populations and additional samples that had been further sorted into subtypes. Data came from three main sources: arrays run and data processed in the Absher lab at HudsonAlpha Institute for Biotechnology (Absher data), Reinius et al. (2012) (Reinius data), and Zilbauer et al. (2013) (Zilbauer data). M450 data from whole blood samples whose cell-type percentages had been quantified was used to calibrate and test the model. Whole blood data consisted of 44 samples from the Absher data and six samples from the Reinius data. Sorted cell data used in model development consisted of 6 CD4+ T cell samples (Zilbauer data), 6 CD8+ T cell samples (Reinius data), 22 CD14+ monocyte samples (Absher data), 62 CD19+ B cell samples (Absher data), 6 granulocyte samples (Reinius data), and 2 natural killer cell samples (Absher data). Samples of sorted T and B cell subtypes were obtained from the Absher data and consisted of 17 naïve CD4+ T cell samples, 18 memory CD4+ T cell samples, and 13 regulatory CD4+ T cell samples, 4 naïve CD8+ T cell samples, 4 memory CD8+ T cell samples, 35 naïve B cell samples, and 64 memory B cell samples. Memory B cell samples consisted of two independently sorted groups, 30 that had undergone isotype class switching and 34 that had not. More information on the data sets can be found in the Supplementary Methods (Section 1 of Supplementary Material) and Supplementary Table 1. All data sets were de-identified prior to inclusion in this work. The project was approved by the IRB as non-human subjects research (IRB Protocol #N140904004).

### Quality control and normalization

Each data set from each study and each cell type was processed independently using the same quality control and normalization pipeline. All data sets were preprocessed from raw beta values, which represent the proportion of methylation at each CpG site for each sample, by first setting any data points to missing in which a significant signal could not be detected as compared to background using a cutoff value of 0.01 for Illumina's detection *p*-value. Next all CpGs with >10% missing data in the data set were removed, and all samples with >1% missing data were removed. Missing values were imputed using the impute.knn function in the impute package in R version 3.1.1 in order to carry out normalization. Data were then batch normalized using the Combat function (Johnson et al., 2007) using subsets of 20,000 CpGs run in parallel to improve computational efficiency. For the purposes of batch correction, a batch was defined as a single array consisting of 12 samples. For smaller data sets in which all samples were run on a single array, the batch normalization step was omitted. Next, samples were normalized to adjust for differences between the Infinium I and Infinium II probe chemistries on the M450 array using a method that fits a polynomial curve to adjacent Infinium I and Infinium II CpGs within 50 bp of one another (Absher et al., 2013). Supplementary Figure 1 displays the results of the normalization method on the global distribution of beta values in comparison to the raw beta values and BMIQ-normalized beta values (Teschendorff et al., 2013), a widely-used normalization method for M450 data. Finally, all missing values were reintroduced into the data sets where imputed values had been positioned prior to normalization.

A principal component analysis (PCA) was conducted using a random subset of 5000 CpGs for all data sets with main cell type data. This was used to determine the best data sets to use for our model in terms of clean clustering and clear separation of one cell type from another. It was also used to find any outliers within a cell-type set and exclude them for the purpose of the analysis. Additional PCA analyses were conducted independently in the same manner for CD4+ T cell subtypes, CD8+ T cell subtypes, and B cell subtypes. For each cell type and subtype, the median of all QC-filtered samples of that cell type for each QC-filtered CpG was calculated and used as the covariate basis for that cell type in the model. For the purpose of model fitting and estimation, all CpGs that contained SNPs within the probe sequence with minor allele frequencies above 0.01 were removed from the data.

### Data simulation

In addition to all of the M450 data sets used, we created simulated data using several of the M450 data sets described above. Simulating whole blood data was necessary because we did not have any whole blood methylation data with measured percentages of T and B cell subtypes. We created 100 simulated “whole blood” mixtures by creating a linear combination of samples with sorted samples of each type and/or subtype using proportions comparable to what would be expected for whole blood. In order to do this, we used a normal distribution to draw a random proportion for each cell type. Parameters for the normal distributions for each cell type are described in Supplementary Table 2. Approximate percentages for each cell type in whole blood were obtained using a chart provided by Stemcell Technologies^1^. Any proportion simulations value < 0 were set to 0. For CD4+ T cells, CD8+ T cells, and B cells, we also simulated proportions of subtypes in order to have known subtype proportions for each of these cells to use in fitting and testing of the models for T and B cell subtypes. Once subtype proportions were determined, the sum was calculated for each of CD4+ T cells, CD8+ T cells, and B cells. The estimates were scaled such that the sum of the subtype percentages for each of these three classes was equal to the simulated percentage value for the respective class (CD4+ T cells, CD8+ T cells, or B cells) for that sample. For the B memory subtype, we simulated percentages for both isotype-class-switched and unswitched B memory cells since our available samples had been sorted into these subsets. For the purposes of model evaluation and testing, we combined the percentages of these two types into a single “B memory” percentage.

Once proportions had been simulated for each cell type, we added the proportions together and scaled them such that the total would sum to 100%. We then randomly selected a single sample from each corresponding sorted cell-type data set to use for each mixture. Once percentages had been determined and random sorted cell samples were selected, a linear combination was created by multiplying the selected proportion for that cell type by the methylation beta values for all CpGs for the selected sample for each cell type and adding up all results for a single sample. We verified that simulated whole blood samples mimicked real whole blood samples by performing a PCA and demonstrating that simulated whole blood samples cluster with real whole blood samples using the first two principal components (Supplementary Figure 2).

Data was simulated in a similar fashion using subtypes of CD4+ T cells to create a sample representative of a sorted CD4+ T cell sample with known subtype proportions. Using the same approach, simulated samples representing CD8+ T cells and B cells were created using a linear mixture of their respective subtypes. For each of the three cell types, 100 simulated samples were created. The simulated proportions of each cell type were based on a normal distribution with parameters described in Supplementary Table 1 using the subtypes of the respective cell type. After simulating proportions of each subtype, the proportions of all corresponding subtypes for each simulated sample were summed and these proportions were scaled such that the total was equal to 1. In the same way as for the “whole blood” simulations, samples were chosen at random from each respective subtype data set. Finally, a linear combination was created by multiplying the beta values in the randomly selected samples of each respective subset by the corresponding simulated proportion.

### Statistical models

Our method uses a linear regression model to estimate cell-type proportions for six major cell types: CD4+ T cells, CD8+ T cells, monocytes, B cells, granulocytes, and natural killer cells. The intercept is removed from the model, and the model is restricted such that all coefficients must be positive and the sum of the coefficients must be ≤ 1. The model is described in Equation 1 below.

(1) $$\begin{array}{l} B=p_{CD\text{4}}X_{CD\text{4}}\text{+}p_{CD\text{8}}X_{CD\text{8}}\text{+}p_{CD\text{14}}X_{CD\text{14}}\text{+}p_{CD\text{19}}X_{CD\text{19}}\\ \text{ +}p_{Gran}X_{Gran}\text{+}p_{NK}X_{NK}\text{+}e \end{array}$$

Here *B* represents the methylation beta values of a mixed sample made up of various cell types, the *X* terms represent the methylation beta values of purified cells of the six main cell types that make up the sample in B (CD4+ T cells [CD4], CD8+ T cells [CD8], CD19+ B cells [CD19], CD14+ monocytes [CD14], granulocytes [Gran], and natural killer cells [NK]), the p terms represent the mixing proportions of the six cell types, and e is the random error term (*e* ~ *N*(0, σ^2^)). We built upon the linear regression model and R function used by Abbas et al. (2009) to implement our method for use with methylation data. The linear regression model was adapted by removing the intercept from the linear regression model and forcing the sum of the coefficients to be ≤ 1. Although there are no obvious violations of linear model assumptions in this setting, the linear model assumptions can be relaxed a bit in this setting since we are only interested in the least-squares coefficients and not the standard errors or hypothesis tests.

In order to run the model to obtain accurate estimates of the mixing proportions, a set of CpGs that most distinctly distinguish cell types was selected for inclusion into the model. First, all CpGs with SNPs at the CpG or within the probe were excluded from consideration. Then, a set of CpGs was chosen based on those that discriminated best between cell types. This lists consisted of two sub-lists. The first sub-list was based on CpGs that have the most significant differences overall for all cell-types using an ANOVA model. This list was chosen based on an ANOVA model fit to methylation data from sorted cells of all the main cell types (CD4+ T cells, CD8+ T cells, monocytes, B cells, granulocytes, and NK cells) using cell type as the grouping variable. The list was ranked in order from the smallest ANOVA *p*-value to the largest, and the top *m* CpGs from this list were used in the deconvolution model. The second sub-list used CpGs that uniquely discriminate one cell type from one other cell type based upon *t*-tests for pairs of cell types. For this sub-list, we chose the top *n* CpGs (based on lowest *t*-test *p*-values) for each of pair of cell types such that these *n* CpGs were not found within the top *n* CpGs from *t*-tests between any other pair of cell types. The sub-list lengths, *m* and *n*, were chosen using an expectation-maximization (EM) algorithm to minimize an error function based on standardized correlation of estimated and measured cell-type percentages and mean squared error (MSE) values using the two whole blood data sets for which blood count data were available (Reinius whole blood data and Absher whole blood data). In order to produce the best possible estimates, accounting for the accuracy and the precision of the estimates using the MSE was important. Furthermore, since cell-type composition estimates are commonly used as covariates in regression models, it was also important to preserve a clear linear relationship with the true estimates as measured by the correlation of the estimated and observed cell compositions. Therefore, we chose an error function that incorporated both the MSE and the correlation between true and predicted values. Details of the EM algorithm and error function can be found in Algorithm 1 in Section 2 of the Supplementary Material. Although the algorithm is complex, we felt it resulted in better model fit than other algorithms we tried that did not include all of these components. We did not explicitly exclude correlated CpGs in the model, so there could be potential violations of independence of observations in the model. However, we did not have any serious concerns about this issue, especially since we only used the coefficient estimates from the model and not the standard errors.

#### Two-stage model

The estimates from the model were further refined for similar cell-types with subtler differences that are more difficult to separate in the main model (for example, a more refined estimate of CD4+ T cells vs. CD8+ T cells) and were then further divided into subsets (such as naïve, memory, and regulatory CD4+ T cells) using a two-stage modeling approach. The first stage of the model was carried out using linear regression using the main model in Equation 1. The methylation profile of *B* was then partitioned into one or more components using an equation of the following form, obtained by rearranging the fixed effect terms in Equation 2, where the $\hat{p}$ terms in the equation below represent the estimates obtained from the main model in Equation 2. *B* in Equation 2 is equivalent to *B* in Equation 1 with the exception that the vectors in the two equations represent a different subset of CpGs as determined by the corresponding CpG selection algorithm (Section 2 of the Supplementary Material).

(2) $$\begin{array}{l} {\hat{B}}_{CD\text{4},CD\text{8}}\approx B-({\hat{p}}_{CD\text{14}}X_{CD\text{14}}\text{+}{\hat{p}}_{CD\text{19}}X_{CD\text{19}}\\ \;+{\hat{p}}_{Gran}X_{Gran}\text{+}{\hat{p}}_{NK}X_{NK}) \end{array}$$

This partitioned beta value was then used as the outcome in a new regression model similar to the one in Equation 1, but containing only the cell types (or subtypes) of interest. For example, for CD4+ T cells vs. CD8+ T cells, the model would be

(3) $$\begin{array}{r} B_{CD4,CD8}=p_{CD4}X_{CD4}+p_{CD8}X_{CD8}+e \end{array}$$

where ${\hat{B}}_{CD4,CD8}$ from Equation 2 is used as an estimate for *B*_*CD*4, *CD*8_. A new set of CpGs was chosen for inclusion in the model in the similar way as for main model in Equation 1, but based on the cell types in question only. With only two cell types in question, CpGs based on overall ANOVA *p*-values were not necessary, so only results from pairwise *t*-tests between the two types in question were used. Since only a single variable had to be optimized (*n* in Algorithm 2 of Section 2 of the Supplementary Material), an EM algorithm was unnecessary to determine the value of this variable that minimized the error function. This simplified CpG selection procedure is described in Algorithm 2 in Section 2 of the Supplementary Material. After *p*_*CD*4_ and *p*_*CD*8_ in Equation 3 were estimated, the estimates were rescaled such that the sum of the resulting estimates, $\left({\hat{p}}_{CD4}+{\hat{p}}_{CD8}\right)$ from Equation 3 was equal to the sum of the corresponding estimates from the main model in Equation 1. This was done so that the second stage refinement did not affect the estimates for other cell types not included in the second stage.

#### Estimating percentages of T and B cell subtypes

The same approach as in the second stage of the two-stage model was applied to estimate subtypes of T and B lymphocytes. For CD4+ T cells, we estimated proportions of the following subtypes: CD4+ T-memory, CD4+ T-naïve, and CD4+ T-regulatory cells. For CD8+ T cells, we estimated proportions of CD8+ T-naïve and CD8+ T-memory cells. Additionally, for B cells, we estimated proportions of naïve B cells and memory B cells (including memory cells that had undergone isotype class switching and those that had not).

The methylation profile can be estimated for the CD4+ T cell population only, using a method analogous to the one in Equation 2. The CD4+ T cell methylation profile for each CpG can then be estimated using the following equation:

(4) $$\begin{array}{l} {\hat{B}}_{CD\text{4}}\approx B-({\hat{p}}_{CD\text{8}}X_{CD\text{8}}\text{+}{\hat{p}}_{CD\text{14}}X_{CD\text{14}}\text{+}{\hat{p}}_{CD\text{19}}X_{CD\text{19}}\\ \;+{\hat{p}}_{NK}X_{NK}\text{+}{\hat{p}}_{gran}X_{gran}) \end{array}$$

where the $\hat{p}$ terms represent the model-estimated percentages of the cell-type referenced in the subscript. These estimates of ${\hat{B}}_{CD4}$ can then be used as the outcome in a new regression model as below to estimate proportions of the T cell subtypes.

(5) $$\begin{array}{r} B_{CD4}=p_{Tmem}X_{Tmem}+p_{Tnaive}X_{Tnaive}+p_{Treg}X_{Treg}+e \end{array}$$

The estimation was carried out in the same way as for the previous models in order to estimate cell-type percentages for each of the T cell subtypes. CpGs were chosen for inclusion into the model using the same method as for the main model, described in Algorithm 1 in Section 2 of the Supplementary Material.

The same methods were used to estimate subtypes for CD8+ T cells and B cells. Since only two subtypes were estimated for these cell types, Algorithm 2 in Section 2 of the Supplementary Material was used to choose the CpGs for these models.

All model development and evaluation was conducted using R version 3.1.1. An R package, called *MethylDeconBloodSubtypes*, which implements our methods is available at https://github.com/HudsonAlpha/MethylDeconBloodSubtypes. To better allow for adaptability of our method to different platforms, including whole genome bisulfite sequencing (WGBS), we provide the R functions used to select CpGs for inclusion in the model as well as the functions needed to fit the model.

## Results

A PCA was conducted using all data sets with sorted samples from main cell types. A plot of the first two principal components is displayed in Supplementary Figure 3. Based on this plot, we chose the most tightly clustered data sets that were the most clearly separated from other cell types to use as a covariate basis in the model. The selected data sets were Zilbauer CD4+ T cells, Reinius CD8+ T cells, Absher CD14+ monocytes, Absher CD19+ B cells, Reinius granulocytes, and Absher NK cells. We also examined this plot for outliers that did not cluster with their expected sample groups. For the data sets selected for inclusion in the model, we did not find any outliers warranting exclusion.

Three similar PCAs were conducted independently for CD4+ T cell subtypes, CD8+ T cell subtypes, and B cell subtypes. Plots of the first two PCs can be seen in Supplementary Figure 4. One PC outlier was found among CD8+ naïve T cell samples, and another outlier was found among CD8+ memory T cell samples. Both of these samples were removed from further analysis.

### Two-stage model for main cell types

Models were run to predict cell-type percentages for CD4+ T cells, CD8+ T cells, CD19+ B cells, CD14+ monocytes, granulocytes, and natural killer cells. The main model described in Equation 1 in Materials and Methods was used to obtain initial estimates for the proportions of all six main cell types. Then, estimates of CD4+ T cell and CD8+ T cells proportions were refined using the second stage of the two-stage model described in Equation 3. We also attempted to refine estimates of monocytes and granulocytes with the second stage of the two-stage model. However, we were unable to obtain estimates that were any better than the main model (first stage) as measured with MSE and correlation using the error function described in Algorithm 1 in Section 2 of the Supplementary Material.

**Figure 1:**
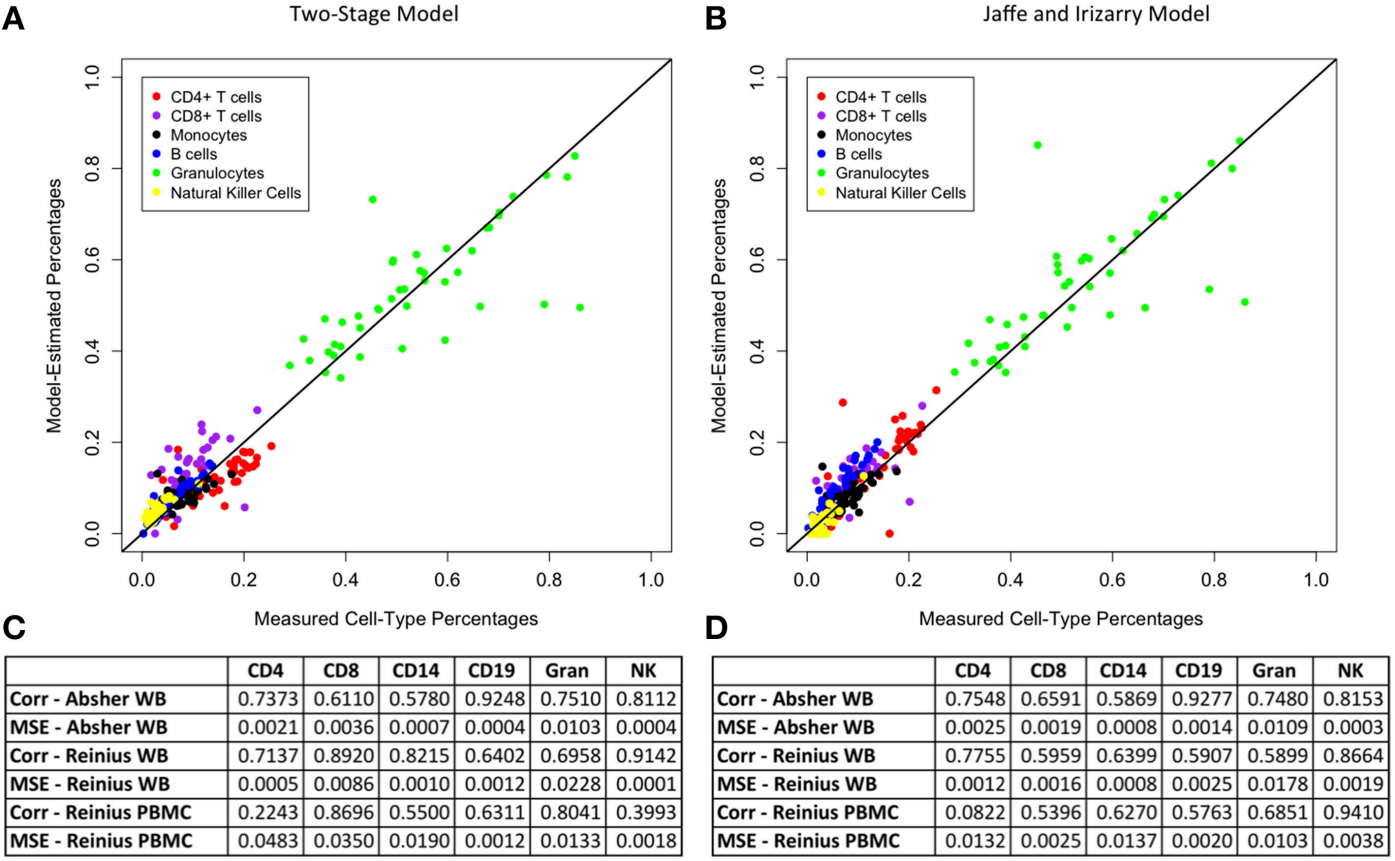
**Comparison of model estimates for main cell types from our two-stage model and the Jaffe and Irizarry model**. Plots of measured vs. model-predicted cell type proportions for each cell type for the Absher whole blood data and associated correlation and MSE for each cell type for each of three data sets (Absher whole blood, Reinius whole blood, and Reinius PBMC) using our two-stage model **(A,C)** and the Jaffe and Irizarry model **(B,D)**.

We compared the results of our two-stage model for main cell-type percentages to those of Jaffe and Irizarry (2014), who developed an updated version of the Houseman et al. (2012) method for M450 data using the function “EstimateCellCounts” in the R package *minfi*. Figure 1 demonstrates a plot of measured vs. model-predicted cell-type proportions for the Absher whole blood data for our two-stage model and the Jaffe and Irizarry model. The figure also contains a comparison table between the performance of our method and the method of Jaffe and Irizarry in terms of MSE and correlations between measured and model-predicted cell-type compositions for each of the six main cell types for three data sets: Reinius whole blood data, Reinius PBMC data, and Absher whole blood data. The performance of our two-stage model is very similar to that of the Jaffe and Irizarry model in terms of correlation and MSE. Although our model performs better for some cell types for some data sets, the Jaffe and Irizarry method performs better for other cell types and/or other data sets. We did not expect our model to perform better than the Jaffe and Irizarry method, since the innovation of our method is not in the estimation of proportions of main cells types but in the estimation of proportions of subtypes of T and B cells.

With regard to our whole blood samples from the Absher autoimmune twin whole blood data set, we were concerned that cell-type percentage estimation accuracy may differ between cases and controls. We compared the difference between CBC-derived cell proportions and the model-predicted cell proportions for cases vs. controls (Supplementary Figure 5). The distributions of these differences were quite similar for cases and controls for each cell type, and there was no evidence of significant differences in the distribution of these differences for any cell type (*p* > 0.05 from the Kolmogorov-Smirnov test for each of the six cell types).

### Model for subtypes of T and B cells

In order to estimate percentages of CD4+ T cell, CD8+ T cell, and CD19+ B cell subtypes, we first partitioned the methylation beta values into the portion representing CD4+ T cells, CD8+ T cells, or B cells respectively (see Section Materials and Methods for details). A regression model using CpGs specific to CD4+ T cell, CD8+ T cell, or B cell subtypes was then fit to this data. For CD4+ T cells, we estimated proportions of T memory, T naïve, and T regulatory cells. For CD8+ T cells, we estimated proportions of naïve and memory T cells. For CD19+ B cells, we estimated proportions of naïve and memory B cells. For the development and calibration of the model, we used simulated whole blood data created by producing a linear combination of sorted cell beta values in proportions mimicking plausible values for whole blood.

**Figure 2:**
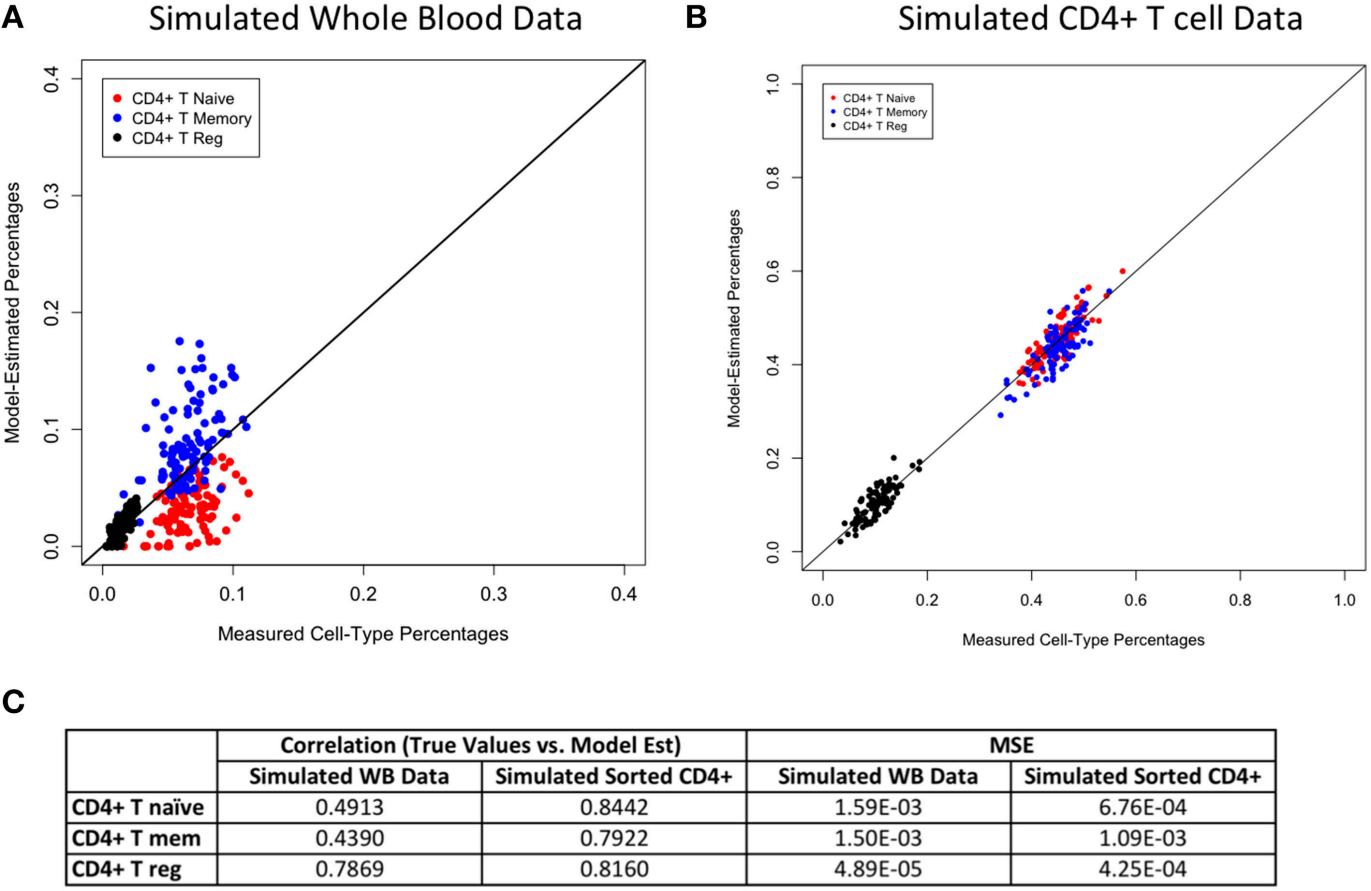
**Model performance for CD4+ T cell subtypes**. True vs. model-estimated proportions for CD4+ T cell subtypes for simulated whole blood data **(A)** simulated sorted CD4+ T cell data **(B)**. Correlations and mean square errors (MSE) of true vs. model-estimated CD4+ T cell subtype proportions **(C)**.

**Figure 3:**
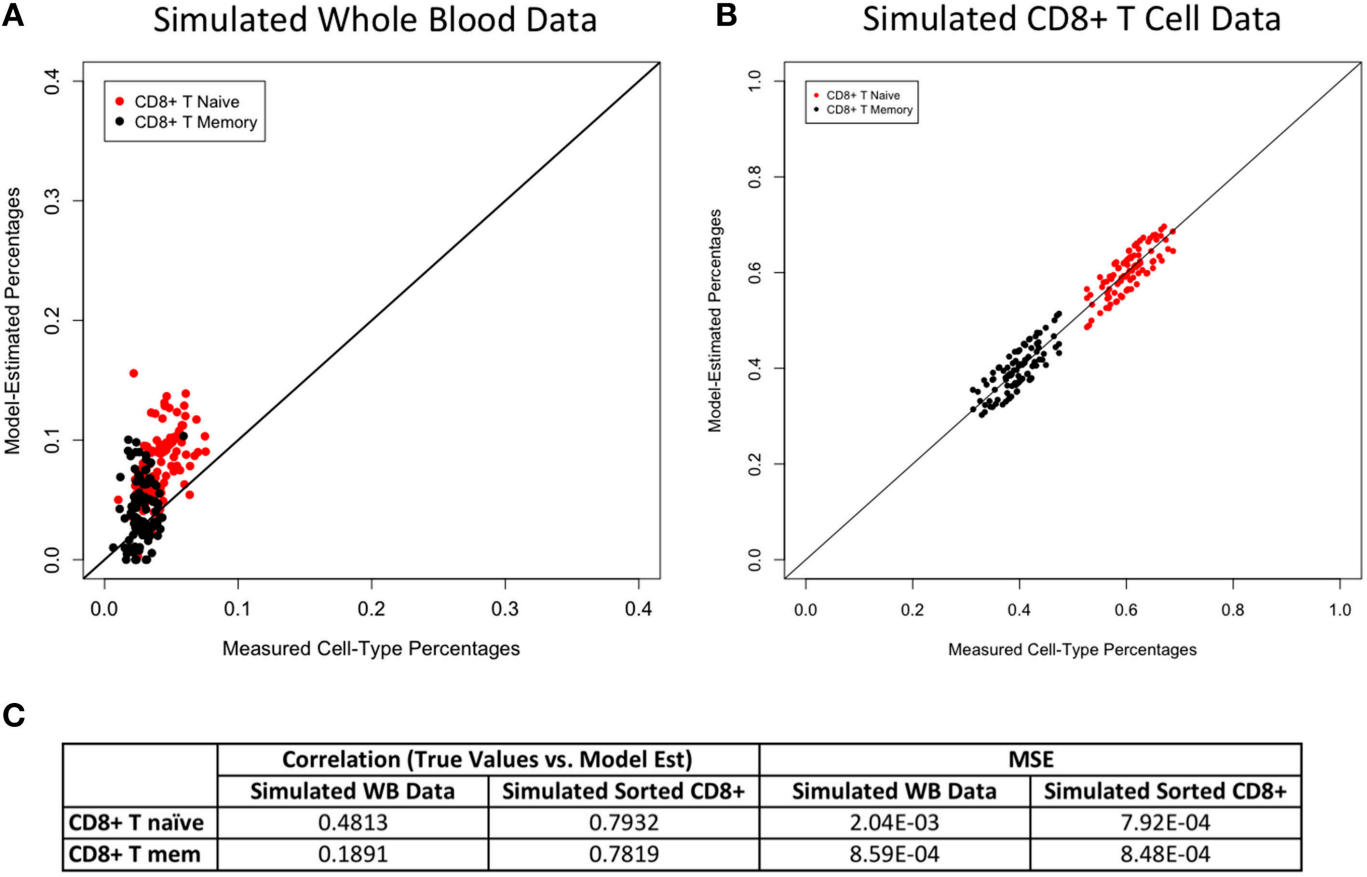
**Model performance for CD8+ T cell subtypes**. True vs. model-estimated proportions for CD8+ T cell subtypes for simulated whole data **(A)** and simulated sorted CD8+ T cell data **(B)**. Correlations and mean square errors (MSE) of true vs. model-estimated CD8+ T cell subtype proportions **(C)**.

**Figure 4:**
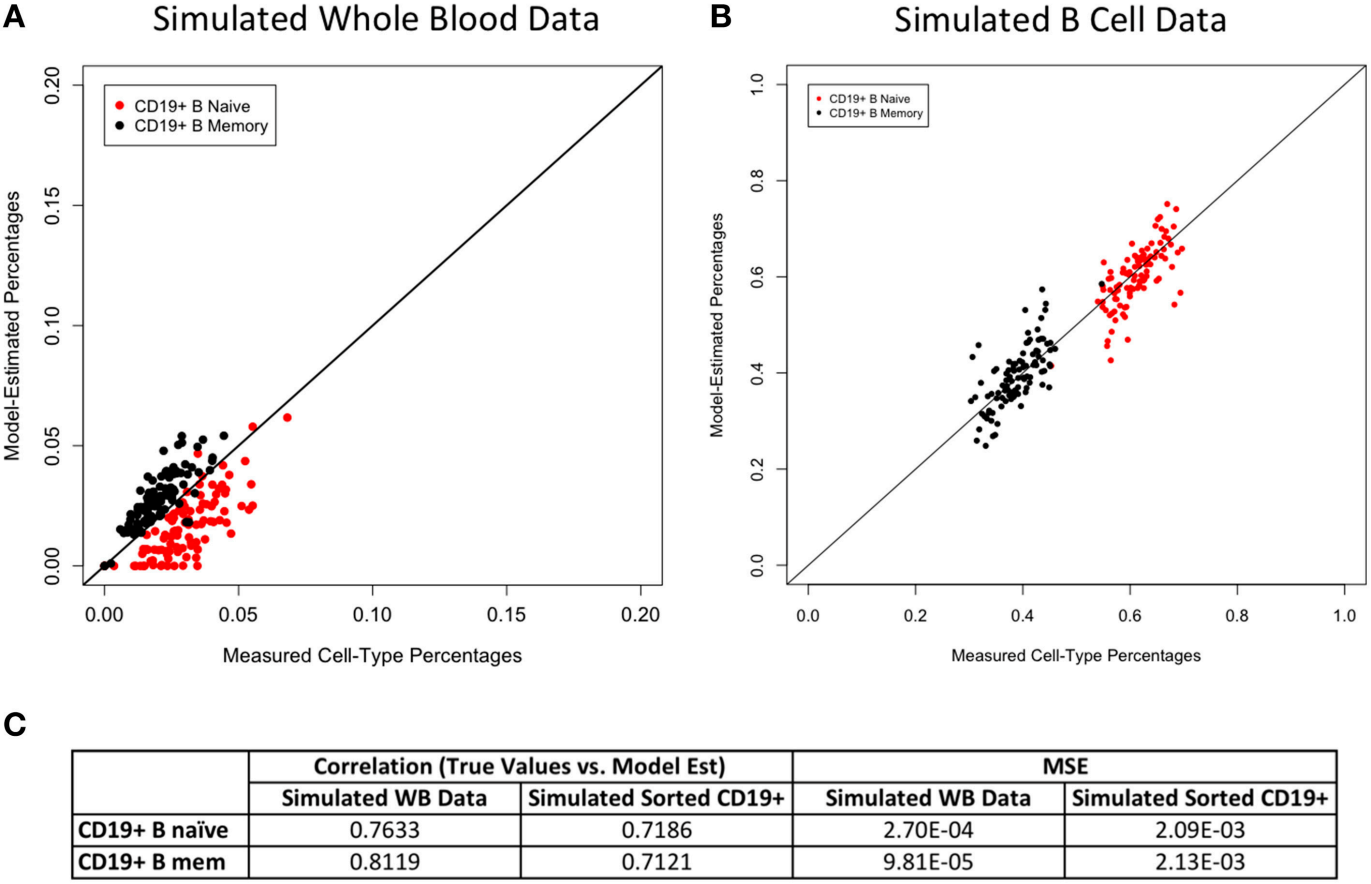
**Model performance for B cell subtypes**. True vs. model-estimated proportions for CD19+ B cell subtypes for simulated whole blood data **(A)** and simulated sorted B cell data **(B)**. Correlations and mean square errors (MSE) of true vs. model-estimated CD19+ B cell subtype proportions **(C)**.

Figures 2–4 display plots of true vs. model-estimated values for proportions of CD4+ T cell, CD8+ T cell, and B cell subtypes, respectively for both simulated whole blood samples and simulated sorted CD4+ T cell, CD8+ T cell, and B cell samples. Additionally, the correlation and MSE of true vs. model-predicted values are displayed for both data sets. The model estimates are quite good for sorted cells, even better than the corresponding estimates for whole blood samples. It is easier to estimate subtype proportions in sorted cells than it is in whole blood because the subtypes make up much larger proportions of the sorted sample, and the total number of cell types in sorted samples is much smaller than in whole blood samples. In whole blood samples, the accuracy of the subtype estimation, as measured by the difference between the model-estimated and true proportions, is strongly associated with the accuracy of the estimation for the corresponding main cell type (CD4+ T cells, CD8+ T cells, or CD19+ B cells; Supplementary Figure 6).

## Discussion

Our method provides a simple technique to estimate the proportion of major cell types in whole blood as well as subtypes of T cells and B cells using DNA methylation array data. Although several authors (Houseman et al., 2012; Jaffe and Irizarry, 2014) have previously provided methods for obtaining estimates for major cell types, and one publication (Marioni et al., 2015) describes the estimation of naïve T cell proportions in whole blood using methylation microarrays, we are the first to estimate multiple proportions of T and B cell subtypes. Our unique methodology provides a process to partition methylation beta values into estimated proportions contributed by specific cell types, which provides a way to obtain precise estimates for T and B cell subtypes. Furthermore, although our model was developed using data from M450 arrays, the methods are easily extendable to WGBS or any other methylation data platform with genome-wide coverage. Our software provides functions to select CpGs to use in the model, which, with the input of WGBS data for sorted cell types, could be easily extended to include CpGs not on the M450 array. Even in the absence of sorted cell WGBS data, one could easily estimate proportions of cell types and subtypes using our method on WGBS whole blood samples using CpGs that overlap with M450 CpGs.

In addition to the added utility of our method in estimating the composition of T and B cell subtypes, our two-stage model differs from previous methods (Houseman et al., 2012; Jaffe and Irizarry, 2014) in that we use a different approach for predicting main cell types. However, the performance of our method for the estimation of cell composition for the main cell types is on par with these methods. It is important to note that the comparison of the two methods is based on using CBC or FACS estimated cell counts as a gold standard. However, these counts are subject to error as well. For example, for CD4+ T cell, cells are typically selected through positive selection for CD4 expression only. Several authors have demonstrated that other cell types, most notably monocytes, express CD4 as well (Gartner et al., 1986; Crowe et al., 1987; Faltynek et al., 1989; Filion et al., 1990). Positive selection for CD4 expression alone (such as with Dynabeads) may not be sufficient to purify T helper cells from other populations that also express CD4 to some degree. Our model uses CD4+ T cell data that has been depleted of monocytes as our covariate basis for estimating CD4+ T cell proportions. Using this CD4+ T cell data as a covariate basis in our model allows for a better estimation of true CD4+ T cell proportions devoid of monocytes. However, since the CD4+ T cell proportion of the all of the whole blood and PBMC data sets used for our model validation were measured without monocyte depletion, our CD4+ T cell estimated proportions do not line up as well with this “gold standard” as they would had the CD4+ T cells been measured after depletion of monocytes.

The ability of our model to estimate proportions of T and B cell subtypes is novel compared to existing methods in this field. We were able to validate our estimates using simulated whole blood methylation data as well as simulated sorted CD4+ T cells, CD8+ T cells, and CD19+ B cells. Our estimates for CD4+ T cell subtypes and B cell subtypes were quite accurate as measured using MSE and correlation of model-estimated and true values. Our estimates for CD8+ T cell subtype proportions were not as good as those for CD4+ T cells or B cells; however, they still demonstrated reasonable accuracy for whole blood sample and were extremely accurate for sorted CD8+ T cells. One possible reason for the shortcomings of the model for CD8+ T cell subtypes is that we were only able to use three samples each of naïve and memory CD8+ T cell for our model development. These samples did not cluster cleanly and tightly together in distinct subclasses in a PCA in the same way that our CD4+ T cell and B cell subtypes did. This could be suggestive of true biological difference among these cell types, as the differences between memory and naïve CD8+ T cells may be subtler than those for subtypes of CD4+ T cells or B cells. However, with access to M450 data for additional sorted memory and naïve CD8+ T cell samples, we would hope to see more distinct clusters in our PCA and therefore better estimates for CD8+ subtype proportions in our model.

Although we validated our model for T and B cell subtypes using simulated whole blood data, we did not have any available whole blood data with measured T and B cell subtype counts to use for validation. Nonetheless, the computationally simulated DNA mixture samples reliably validated our model. Because the simulations were based on linear combinations of real data from sorted cells, they closely represented real whole blood samples and clustered with real whole blood samples in a PCA (Supplementary Figure 2). In the future, if methylation data became available for whole blood samples with measured proportions of T and B cell subtypes, we would like to validate our model using real whole blood samples.

Our model provides a unique functionality in estimating proportions of T and B cell subtypes in whole blood and sorted T and B cell samples. Despite a few limitations, we are able to provide reliable estimates of cell-type and subtype proportions for whole blood or sorted B or T cell methylation data. Our estimates of subtype proportions in sorted CD4+ T cells, CD8+ T cells, and B cells are quite accurate. Predicting of subtype proportions in whole blood is a more difficult task. However, our estimates still achieve small values of MSE and large correlations of model-predicted vs. true values. This is impressive given the difficulty of estimating the very small proportions of cell subtypes in whole blood. Our model provides a new functionality that will be valuable in a wide variety of applications for methylation data for whole blood or sorted blood cells.

## Author contributions

LW was involved in developing the method, completing the statistical analysis, producing the results, and writing the manuscript. BW was involved in completing the statistical analysis and writing the R package to implement the method and contributed to the writing of the manuscript. KD was involved in developing the method and writing the manuscript. XL, KR, AG, JE, and RK conceived of and conducted the experiments necessary to obtain the data that was crucial to the development of this method as well as contributed to the writing of the manuscript. DA and HT conceived of the idea for the method and were involved in writing the manuscript.

## Funding

Some of the sorted cell data sets used in this work were funded by the UAB Rheumatic Diseases Core Center (P30-AR48311) and the Center for Clinical and Translational Science (UL1 TR001417). The funders had no role in study design, data collection and analysis, decision to publish, or preparation of the manuscript.

### Conflict of interest statement

The authors declare that the research was conducted in the absence of any commercial or financial relationships that could be construed as a potential conflict of interest.

## Footnotes

^1^http://www.stemcell.com/~/media/Files/wallchart_CellTypes_WEB.pdf.
