# Supplementary material for: Estimation of Cell-Type Composition Including T and B Cell Subtypes for Whole Blood Methylation Microarray Data
Source: Front Genet. 2016 Feb 18;7:23. doi: 10.3389/fgene.2016.00023 (PMC4757643; doi:10.3389/fgene.2016.00023)
Supplement: Supplementary file 1 [file DataSheet1.PDF]

# *Supplementary Material*

# Estimation of cell-type composition including T and B cell subtypes for whole blood methylation microarray data

**Lindsay L. Waite[1,2], Benjamin Weaver[2], Kenneth Day[2], Xinrui Li[3], Kevin Roberts[2], Andrew W. Gibson[3], Jeffrey C. Edberg[3], Robert P. Kimberly[3], Devin M. Absher[2#], Hemant K. Tiwari[1#]***

# These authors share senior authorship on this work.

*** Correspondence:** Dr. Hemant K. Tiwari: htiwari@uab.edu

## 1 Supplementary Methods – Data set descriptions

Six whole blood samples came from healthy controls in Reinius *et al*. (2012). For each of the six donors, M450 data was obtained for whole blood, peripheral blood mononuclear cells (PBMCs), CD4+ T cells, CD8+ T cells, CD19+ B cells, CD14+ monocytes, granulocytes, and natural killer cells. Cell-composition estimates of the whole blood and PBMC samples were obtained using flow cytometry. Raw beta values, representing the proportion of methylation for each sample at each CpG site, were obtained using the R Bioconductor package *FlowSorted.Blood.450k* (Jaffe and Irizarry, 2014) and detection p-values were downloaded from the gene expression omnibus (GEO accession number: GSE35069).

M450 data for an additional 6 CD4+ T cell samples were obtained using data from Zilbauer *et al.* (Zilbauer et al., 2013; Lyons et al., 2007). We chose to include these samples because the cells were sorted first using positive selection for CD14+ monocytes, and then the remaining CD14- portion was sorted using positive selection for CD4+ T cells. This led to a CD4+ T cell population that was depleted in monocytes, a potential advantage over CD4+ T cells from the Reinius and Absher data sets, which were obtained by positive selection for CD4 only. Data were obtained through the ArrayExpress database (accession number: E-MTAB-2145).

The Absher M450 array data came from several different data sets from a few different studies. Forty-four whole blood samples originated from an autoimmune study using 22 autoimmune-discordant monozygotic twin pairs (obtained from Fredrick W. Miller, NIH Clinical Research Center) with available complete blood count (CBC) with differential data, which quantified the percentages of all of the main cell types used in our model (CD4+ T cells, CD8 + T cells, monocytes, B cells, granulocytes, and natural killer cells). Additional data was obtained from 10 controls from an asthma study, including 10 CD4+ T cell samples, 10 CD19+ B cell samples, and 10 granulocyte samples. CD4+ T cells and CD19+ B cells were isolated in parallel from whole blood through positive selection using antigen-specific Invitrogen Dynabeads for CD4 and CD19 respectively, following the manufacturer's protocol. Granulocytes were isolated from anti-coagulated peripheral blood by the Ficoll-Hypaque method. Absher natural killer cell samples consisted of two samples purchased from Astarte Biologics. The cells were sorted using negative selection.

Additional Absher sorted cell samples were obtained from controls in a study of systemic lupus erythematosus (SLE) (Absher et al., 2013), including 52 CD4+ T cell samples, 52 CD19+ B cell samples, and 22 CD14+ monocyte samples. These cells were sorted in parallel utilizing 5ml of whole blood using positive selection with CD4, CD19, and CD14 antigen-specific Invitrogen Dynabeads, following the manufacturer's protocol. CD4+ subtype samples including 18 CD4+ T memory cell samples (CD45RO+RA-), 17 CD4+ T naïve cell samples (CD45RA+RO-), and 13 CD4+ T regulatory cell samples (CD25+CD127-) also came from this study. These cells were sorted via flow cytometry (FACSAriaII, BD Biosciences) using positive selection with anti-CD4-Alexa488, anti-CD45RO-APC, anti-CD45RA-PE, anti-CD25-PerCP- Cy5.5 and anti-CD127-Pacific Blue antibodies (Biolegend, Inc).

Additional samples in the Absher data sets came from CD8+ T cell subtypes and CD19+ B cell subtypes obtained from controls in another SLE study (manuscript in preparation). CD8+ T cells were obtained by positive selection for CD8 expression using Dynabeads. Then, 4 CD8+ memory T cell samples (CD45RO+RA-) and 4 CD8+ naïve T cell samples (CD45RA+RO-) were sorted from the CD8+ T cell populations using flow-cytometry in a method analogous to that for naïve and memory CD4+ T cells as described above. B cell subtypes, including 35 naïve B cell samples ($CD19^{+}CD27^{-}IgD^{+}$), 30 isotype-class-switched B memory cell samples ($CD19^{+}CD27^{+}IgD^{-}$), and 34 unswitched B memory cell samples ($CD19^{+}CD27^{+}IgD^{+}$), were also sorted by flow cytometry.

Six DNA mixture samples were also included as a part of the Absher data sets. These samples were created by mixing pre-specified proportions of DNA from CD14+ monocytes, granulocytes, natural killer cells, CD4+ naïve T cells, CD4+ memory T cells, CD4+ regulatory T cells, CD8+ naïve T cells, CD8+ memory T cells, CD19+ naïve B cells, CD19+ switched memory B cells, and CD19+ unswitched memory B cells. DNA from each respective cell type was obtained from remaining DNA from the sorted cell data sets described above.

For each Absher data set, DNA was extracted using QIAGEN DNAeasy kits. Samples were treated with sodium bisulfite (Zymo EZ-96 DNA Methylation kit), then were run on M450 arrays using the standard Illumina protocol for amplification, hybridization, and imaging. The resulting data was obtained using Illumina's GenomeStudio software to obtain beta values and detection p-values without using any of Illumina's background subtraction or normalization options.

## 2 CpG selection algorithms for inclusion into models

**Algorithm 1 (EM algorithm used for selection of CpGs for models with more than two classes of cell types)**

1. Select starting values for *m* (number of CpGs chosen from overall ANOVA results) and *n* (number of CpGs chosen for each unique pairwise test between two classes of CpGs). We used 1000 as the starting value for both *m* and *n*.
2. Fix *m* and iterate over values of *n.* We used values from 100 to 15,000 in increments of 100. Complete steps 3-9 for each iteration.

3. Choose the top *m* CpGs from the overall ANOVA list based upon ranking the list in ascending order by *p*-value.
4. Create unique pairwise CpG lists for each possible pairwise combination by first ranking the results of the *t*-test for that pair of cells in order of ascending *p*-values. Then remove from the list any CpGs that fall in the top *n* for any other test based on a different pair of cell types. Then choose the top *n* CpGs from each unique pairwise list.
5. Fit the model using the set of CpGs determined by *m* and *n* in steps 3 and 4.
6. Calculate Pearson's correlation between the model-predicted estimates and the true (measured) values for each cell type proportion.
7. Add up the correlation values from all cell types. If more than one data set is being used for model selection, add up the sums from each data set.
8. Compute mean squared error (MSE) for each cell type using the model-predicted estimates and the true (measured) values using the following formula:
$$MSE = \sum_{i=1}^{n} \frac{(\hat{Y}_{model} - Y_{measured})^2}{n}$$
9. Add up the MSE values from all cell types. If more than one data set is being used for model selection, add up the sums from each data set.
10. Once all iterations are complete, standardize the correlation and MSE sums obtained from each iteration in steps 7 and 9 respectively by creating a Z-score (subtract mean value over all iterations and divide by standard deviation over all iterations).
11. Add the standardized MSE from step 10 and the negative of the standardized correlation from step 10 (used such that MSE is minimized and correlation is maximized) together to create the values of the error function.
12. Choose the value of *n* that minimizes the error function in step 11.
13. Fix *n* at the value selected in step 12 and iterate over values of *m*. We used values of *m* from 100 to 50,000 in increments of 100. Repeat steps 3-9 for each iteration.
14. Repeat steps 9-11 and select the value of *m* that minimizes the error function in step 11.
15. Fix *m* at the value selected in step 14 and repeat steps 2-14 until convergence. Check plot of error function to ensure the global minimum was found.

**Algorithm 2 (algorithm used for selection of CpGs for models with only two classes of cell types)**

1. Iterate over values of *n*. We used values from 100 to 15,000 in increments of 100. Complete steps 2-7 for each iteration.
2. Choose the top *n* CpGs based upon the ranking of the results of the *t*-test between the two cell types by from smallest to largest *p*-value.
3. Fit the model using the set of CpGs determined by *n* in step 2.
4. Calculate Pearson's correlation between the model-predicted estimates and the true (measured) values for each cell type proportion.
5. Add up the correlation values from all cell types. If more than one data set is being used for model selection, add up the sums from each data set.
6. Compute mean squared error (MSE) for each cell type using the model-predicted estimates and the true (measured) values using the following formula:
$$MSE = \sum_{i=1}^{n} \frac{(\hat{Y}_{model} - Y_{measured})^2}{n}$$

7. Add up the MSE values from all cell types. If more than one data set is being used for model selection, add up the sums from each data set.
8. Once all iterations are complete, standardize the correlation and MSE sums obtained from each iteration in steps 5 and 7 respectively by creating a Z-score (subtract mean value over all iterations and divide by standard deviation over all iterations).
9. Add the standardized MSE from step 8 and the negative of the standardized correlation from step 8 (used such that MSE is minimized and correlation is maximized) together to create the values of the error function.
10. Choose the value of $n$ that minimizes the error function in step 9. Check plot of error function to ensure the global minimum was found.

## 3 Supplementary Figures and Tables

### 3.1 Supplementary Figures

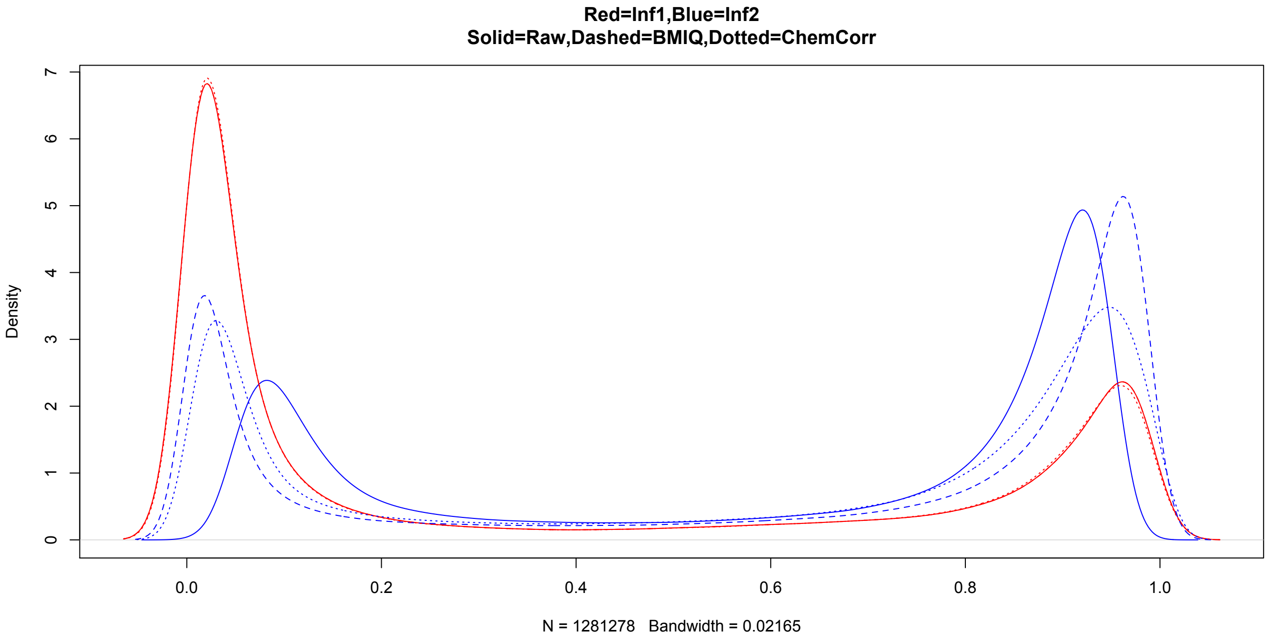


**Supplementary Figure 1.** Density plot of beta values for Infinium I and Infinium II chemistry probes on the Illumina M450 array, showing an example of the chemistry correction normalization used in our QC and normalization pipeline in comparison to raw values as BMIQ normalization (Teschendorff et al., 2013).

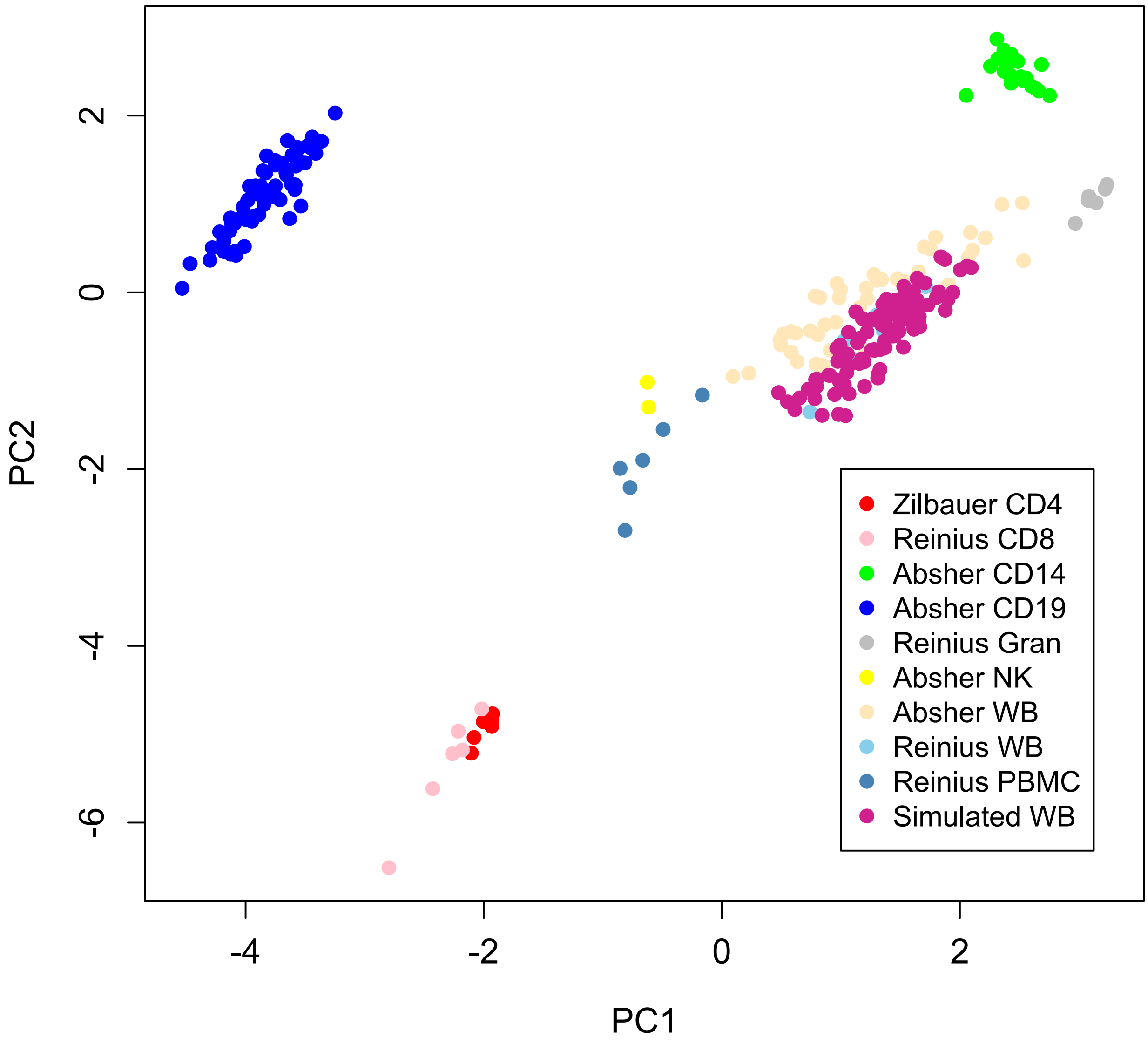


**Supplementary Figure 2.** Plot of the first two principal components from a principal component analysis of the simulated whole blood data with real data from whole blood and sorted cell types. The simulated whole blood data clusters with real whole blood data based on the first two principal components.

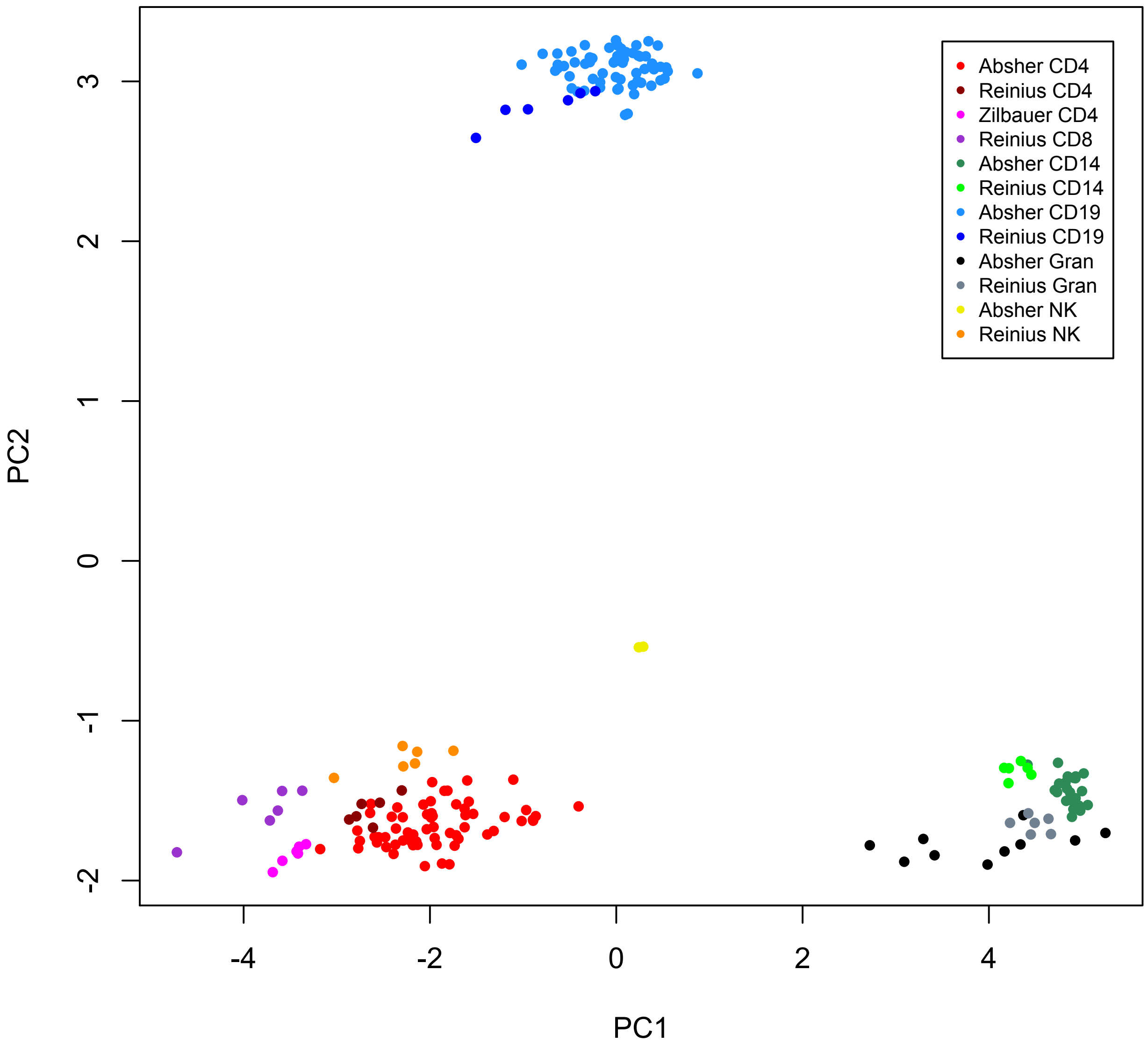


**Supplementary Figure 3.** Principal component 1 versus principal component 2 for all sorted main cell-type samples, color-coded by data set.

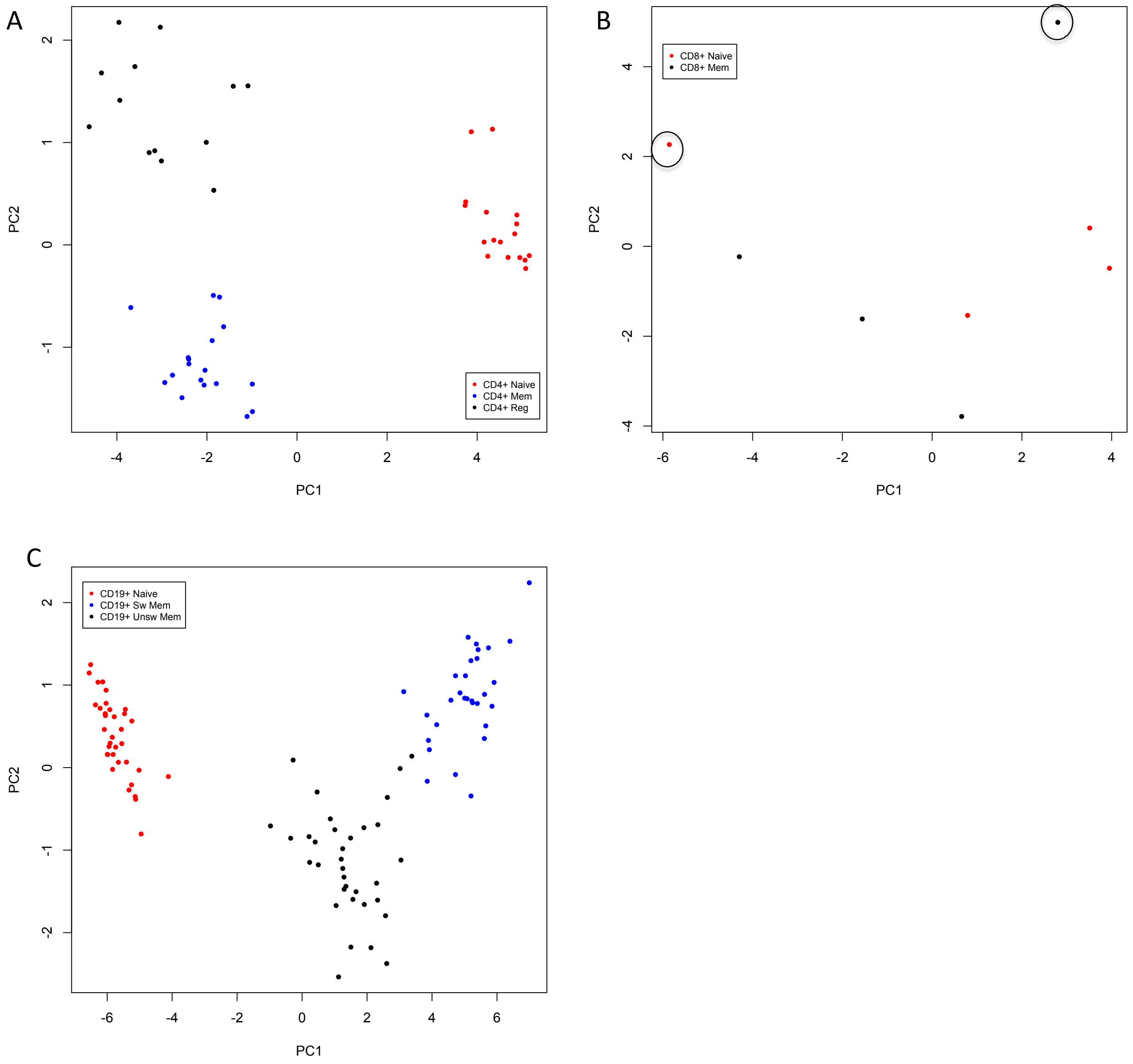


**Supplementary Figure 4.** Plots of the first two principal components for sorted samples of CD4+ T cell subtypes (A), CD8+ T cell subtypes (B), and CD19+ B cell subtypes (C). The circled points in (B) are outliers that were excluded from further analysis.

**Controls**

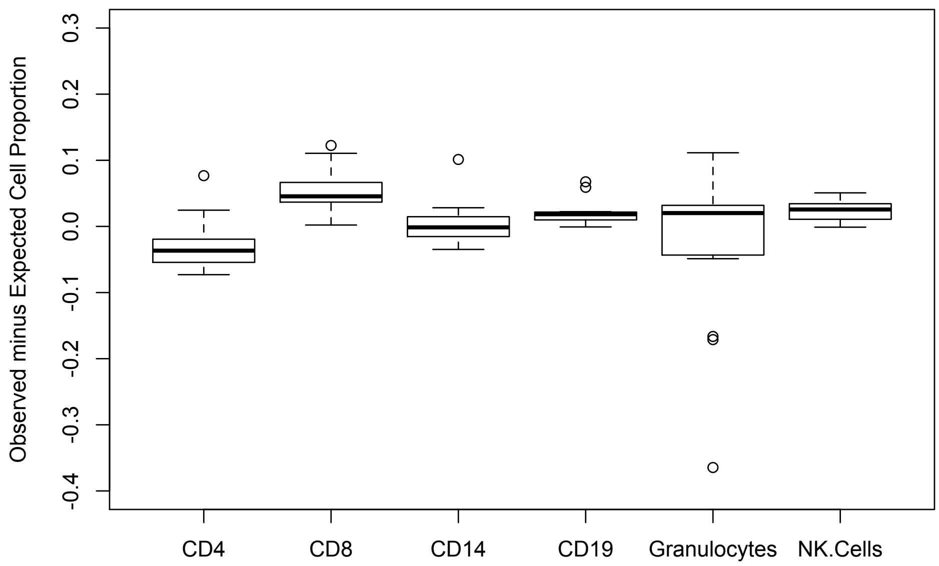


**Cases**

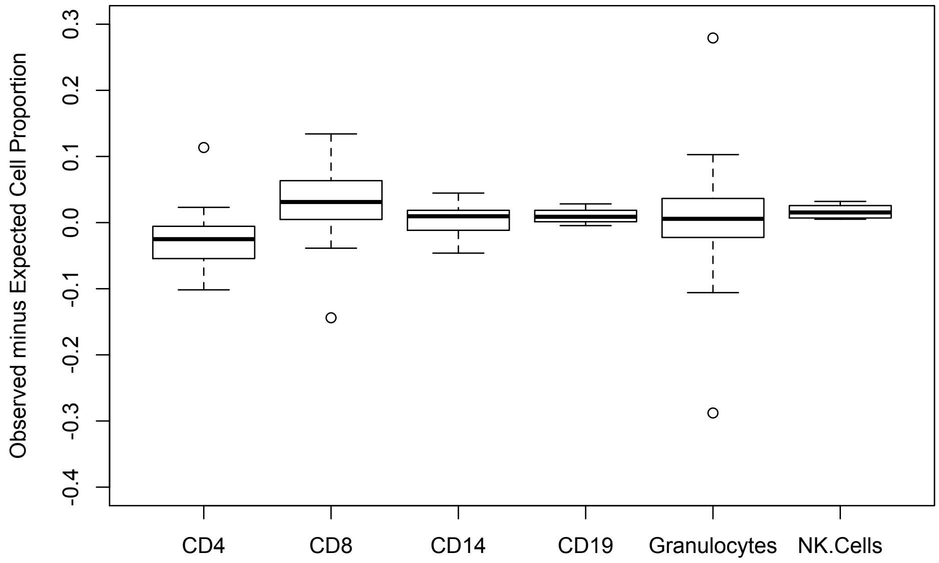


**Supplementary Figure 5.** Observed minus expected cell-type proportions for controls (top) and cases (bottom) in the Absher autoimmune twin whole blood data set. There was no significant difference in the distribution of these values between cases and controls for any of the six main cell types ($p > 0.05$ from the Kolmogorov-Smirnov test for each cell type).

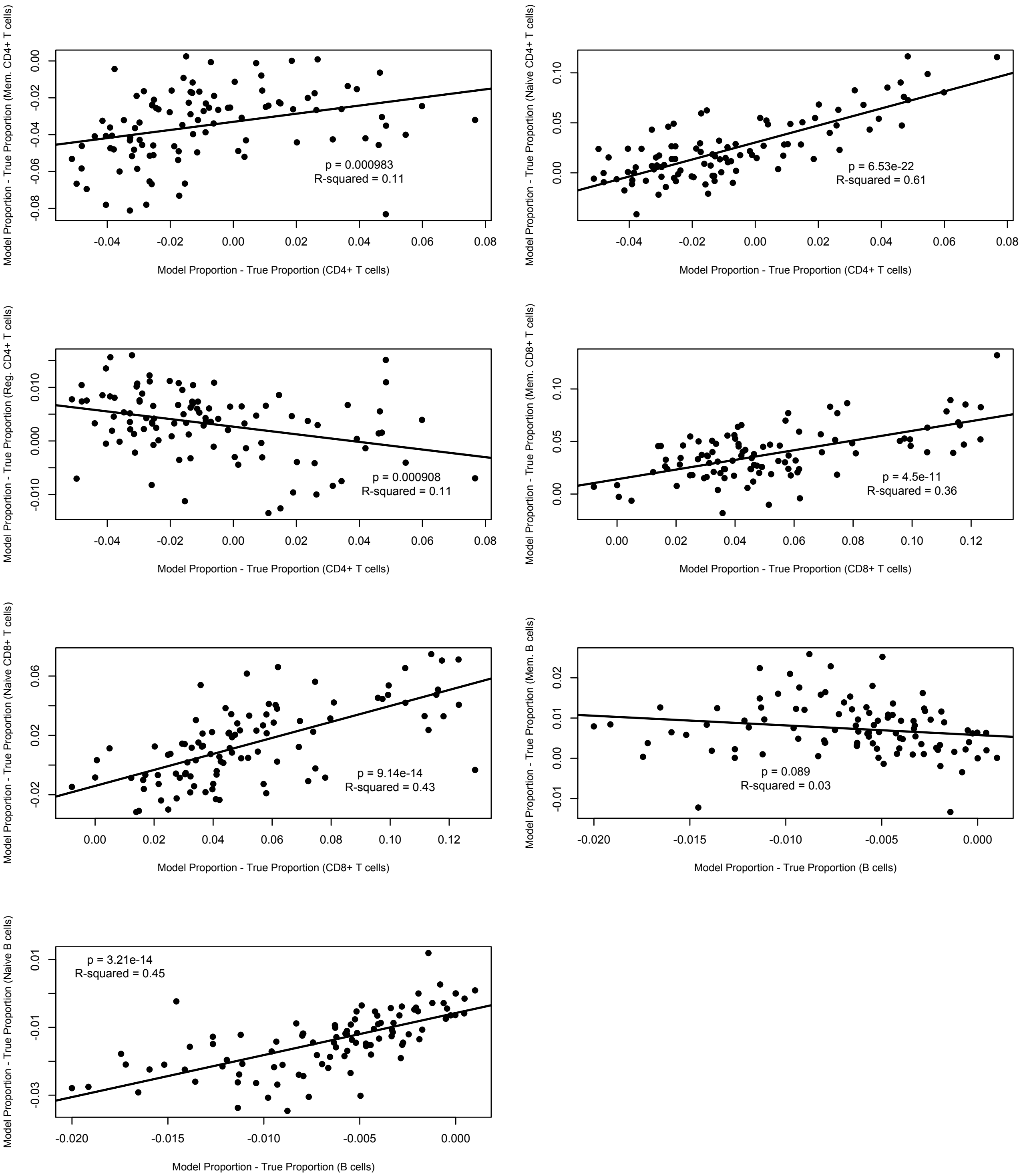


**Supplementary Figure 6.** Error, as measured by the difference between model-predicted and true proportions, of T and B cell subtype estimation versus error of estimation of the corresponding main cell type (CD4+ T cells, CD8+ T cells, or CD19+ B cells).

## 3.2 Supplementary Tables

**Supplementary Table 1.** Number of samples by data set and cell type. The data set for each type that was used in model development is highlighted in yellow.

| Cell Type | Absher data | Reinius Data | Zilbauer Data |
|---|---|---|---|
| Whole Blood | 44 | 6 | N/A |
| CD4+ T cells | 62 | 6 | 6 |
| CD8+ T cells | N/A | 6 | N/A |
| CD14+ Monoctyes | 22 | 6 | N/A |
| CD19+ B cells | 62 | 6 | N/A |
| Granulocytes | 10 | 6 | N/A |
| Natural Killer Cells | 2 | 6 | N/A |
| CD4+ T memory | 17 | N/A | N/A |
| CD4+ T naive | 18 | N/A | N/A |
| CD4+ T regulatory | 13 | N/A | N/A |
| CD8+ T memory | 4 | N/A | N/A |
| CD8+ T naive | 4 | N/A | N/A |
| CD19+ B Naive | 35 | N/A | N/A |
| CD19+ B Switched Memory | 30 | N/A | N/A |
| CD19+ B Unswitched Memory | 34 | N/A | N/A |

**Supplementary Table 2.** Simulation parameters for simulating "whole blood" samples using linear combinations of methylation profiles for sorted cell types and subtypes. Simulated samples of CD4+ T cells, CD8+ T cells, and CD19+ B cells were also created using only subtypes of the respective cell type.

| Cell Type | Cell Subytpe | Normal Dist. Mean | Normal Dist. SD |
|---|---|---|---|
| CD4+ T cells | | 14 | 4 |
| | CD4+ T Naïve | 45 | 5 |
| | CD4+ T Memory | 45 | 5 |
| | CD4+ T reg | 10 | 3 |
| CD8+ T cells | | 7 | 2 |
| | CD8+ T Naïve | 60 | 5 |
| | CD8+ T Memory | 40 | 5 |
| Monocytes | | 5 | 1.5 |
| B cells | | 5 | 2 |
| | B Naïve | 60 | 5 |
| | B Switched Memory | 20 | 4 |
| | B Unswitched Memory | 20 | 4 |
| Granulocytes | | 65 | 7 |
| NK cells | | 2 | 0.5 |
